# Impact of the COVID‐19 pandemic on patients with bipolar disorder in Japan

**DOI:** 10.1002/pcn5.82

**Published:** 2023-03-02

**Authors:** Yuko Isogaya, Dai Kezuka, Chiho Suzuki, Shingo Hoshina, Eiji Suzuki

**Affiliations:** ^1^ Department of Psychiatry Tohoku Medical and Pharmaceutical University Hospital Sendai Japan; ^2^ The Japanese Alliance of Bipolar Disorder Tokyo Japan; ^3^ Division of Psychiatry Tohoku Medical and Pharmaceutical University Sendai Japan

The COVID‐19 pandemic has impacted patients with various psychiatric disorders, including increasing the prevalence of depressive symptoms.^1^ In particular, patients with bipolar disorder (BD) have been reported to experience stronger psychological distress than healthy controls or patients with other psychiatric disorders.^2^, ^3^, ^4^ In patients with BD, the lockdown was found to worsen manic and other symptoms.^4^, ^5^ However, curfew restrictions in Japan have not been as strict as those in Western countries.^6^ Here, we investigated the impact of the COVID‐19 pandemic on Japanese patients with BD.

From January to March 2022, we conducted a questionnaire survey among 528 members of the Japanese Alliance of Bipolar Disorder,^7^ a self‐help group for BD. The survey was conducted after receiving ethical approval from the Clinical Research Review Committee of Tohoku Medical and Pharmaceutical University Hospital. Of the 235 members who agreed to participate in the survey and responded to the questionnaire, excluding 55 responses from family members and related persons, 180 patients with BD (101 women, 66 men, and 13 unknown; self‐reported diagnosis) were included in the analysis. Fisher's exact test was used for statistical analysis using EZR software.^8^

The impact of the COVID‐19 pandemic on overall BD disease status is shown in Figure 1a (see Supporting Information Figure S1 for questions). The response rates for very much worse, worse, a little worse, no impact, a little better, and better were 4.5%, 9.5%, 23.5%, 53.6%, 6.1%, and 2.8%, respectively. Depression (35.6%), irritability (21.7%), and decreased motivation (21.1%) were the top three individual symptoms that were most exacerbated by the COVID‐19 pandemic (see Supporting Information Table S2 for details). A total of 75.6% respondents reported that the COVID‐19 pandemic had some impact on their lives, with the most common impact being increase in stress from not going out (SNG) (38.3%) and increase in time spent on social networking sites (27.8%). Among the effects on life, only increase in SNG was significantly associated with worsening of overall BD disease status due to the COVID‐19 pandemic (Figure 1a; P < 0.0000000001). When the association between increase in SNG and worsening of individual symptoms was examined, a significantly higher proportion of respondents in the group with SNG reported worsening depression (P < 0.000000001), irritability (P < 0.00001), difficulty concentrating (P < 0.0001), and decreased motivation (P < 0.001) (Figure 1b).

**Figure 1:**
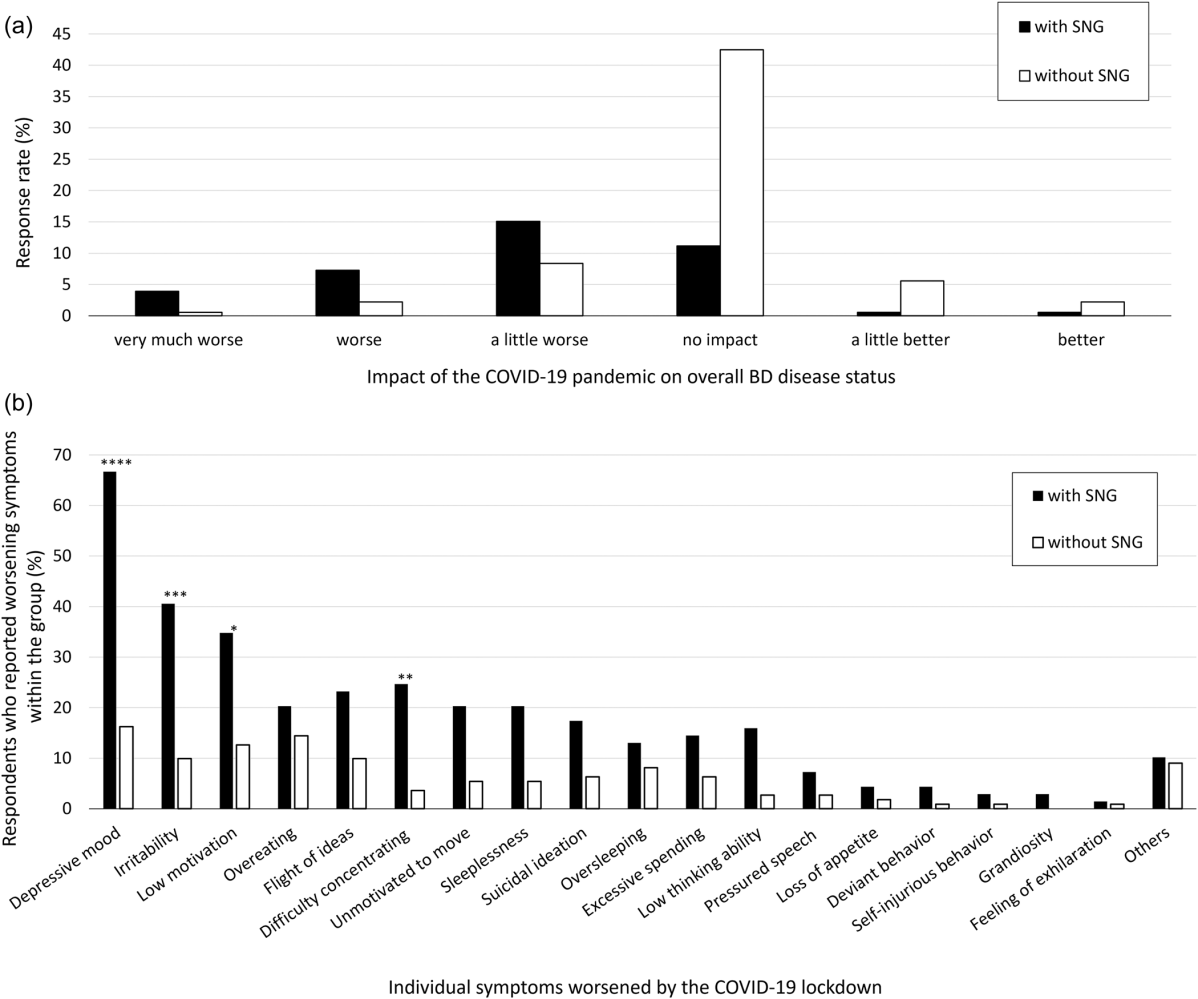
Association between the presence/absence of stress from not going out (SNG) and the impact of the COVID‐19 pandemic on (a) overall bipolar disorder (BD) disease status and (b) individual symptoms. (a) Responses on the impact of the COVID‐19 pandemic on overall BD disease status showed significant differences between the groups with SNG and without SNG. (b) The group with an increase in SNG reported four significantly worsened individual symptoms. Individual symptoms are sorted from left to right in the order of the number of responses. BD, bipolar disorder; SNG, stress from not going out. ****P < 0.000001, ***P < 0.00001, **P < 0.0001, *P < 0.001.

In a survey conducted in Australia in April 2020 during a strict lockdown, 76.9% of patients with BD reported a negative impact on mental health due to government regulations.^3^ Although it may be difficult to make comparisons due to the large differences between the questions asked in that study and our study, only 37.5% of patients in our study reported that the COVID‐19 pandemic had worsened their overall BD disease status. Studies in several European countries, among the general population, have reported that the more severe the lockdown, the worse the mental health (including worry, loneliness, and anxiety).^9^, ^10^ The relatively lower worsening of overall BD disease status may be because the only request made to the public in Japan was to refrain from unnecessary outings (no penalty). In our study, patients who experienced an increase in SNG reported worsening in various symptoms compared to those who did not experience an increase (Figure 1b). These results suggest that for patients with BD, the intensity of curbs on going out was associated with worsening symptoms. Interestingly, while previous studies have reported that lockdown had worsened manic symptoms,^4^, ^5^ the present results suggest that SNG is also associated with worsening of depressive symptoms, including depression and decreased motivation (Figure 1b).

The study results should be interpreted after considering the following factors. This study was conducted 2 years after the start of the pandemic, which was at a time when people were somewhat accustomed to life in the pandemic. The difference in results between previous studies and this study may be due to the passage of time after the pandemic. Furthermore, here, 60% of the respondents who reported that their symptoms had improved due to the COVID‐19 pandemic (16.7%) had positively attributed this improvement to their refraining from going out (e.g., socializing less made them feel more comfortable and the pandemic was a reason to avoid going out in excess).

This study is a cross‐sectional questionnaire‐based survey with no control group, which may have methodological limitations. Nevertheless, it will be beneficial if future studies conduct similar surveys, while including a control group or other disorders, amid the protracted COVID‐19 pandemic to determine how COVID‐19‐related policies, such as requests to refrain from going out, affect patients with BD.

## AUTHOR CONTRIBUTIONS

Eiji Suzuki and Yuko Isogaya designed the study, collected, analyzed, and interpreted the data, and prepared and edited the manuscript. Chiho Suzuki collected the data and edited the manuscript. Shingo Hoshina and Dai Kezuka analyzed and interpreted the data and prepared and edited the manuscript. All authors approved the final manuscript as submitted.

## CONFLICT OF INTEREST STATEMENT

The authors declare no conflict of interest.

## ETHICS APPROVAL STATEMENT

This study was conducted after obtaining approval from the Clinical Research Review Board at Tohoku Medical and Pharmaceutical University Hospital (research registration no.: 2021‐2‐081). The study was explained in writing at the time of the mail survey and written informed consent was obtained from the participants. Participants could withdraw their consent at any time without prejudice.

## PATIENT CONSENT STATEMENT

Informed consent has been obtained from all individuals included in this study.

## CLINICAL TRIAL REGISTRATION

N/A

## Supporting information

Supporting information.

## Data Availability

The data that support the findings of this study are available from the corresponding author, Eiji Suzuki, upon reasonable request.

## References

[1] Ettman CK , Abdalla SM , Cohen GH , Sampson L , Vivier PM , Galea S . Prevalence of depression symptoms in US adults before and during the COVID‐19 pandemic. JAMA Network Open. 2020;3:e2019686. 32876685 10.1001/jamanetworkopen.2020.19686 PMC7489837

[2] Yocum AK , Zhai Y , McInnis MG , Han P . Covid‐19 pandemic and lockdown impacts: a description in a longitudinal study of bipolar disorder. J Affect Disord. 2021;282:1226–33. 33601700 10.1016/j.jad.2021.01.028 PMC9754803

[3] Van Rheenen TE , Meyer D , Neill E , Phillipou A , Tan EJ , Toh WL , et al. Mental health status of individuals with a mood‐disorder during the COVID‐19 pandemic in Australia: initial results from the COLLATE project. J Affect Disord. 2020;275:69–77. 32658826 10.1016/j.jad.2020.06.037 PMC7331562

[4] Schönthaler EMD , Dalkner N , Ratzenhofer M , Fleischmann E , Fellendorf FT , Bengesser SA , et al. Greater emotional distress due to social distancing and greater symptom severity during the COVID‐19 pandemic in individuals with bipolar disorder: a multicenter study in Austria, Germany, and Denmark. Int J Environ Res Public Health. 2022;19:7626. 35805284 10.3390/ijerph19137626 PMC9265390

[5] Koenders M , Mesbah R , Spijker A , Boere E , de Leeuw M , van Hemert B , et al. Effects of the COVID‐19 pandemic in a preexisting longitudinal study of patients with recently diagnosed bipolar disorder: indications for increases in manic symptoms. Brain Behav. 2021;11:e2326. 34554650 10.1002/brb3.2326 PMC8613426

[6] Cabinet Secretariat . Basic policies for novel coronavirus disease control by the Government of Japan (Summary). Cabinet Secretariat; March 28, 2020. Accessed April 16, 2020. https://www.mhlw.go.jp/content/10900000/000624195.pdf

[7] Isogaya Y , Suzuki C , Hoshina S , Nibuya M , Suzuki E . The analysis of telephone consultation contents of patients with bipolar disorder received by a self‐help group. Psychiatry Clin Neurosci Rep. 2022;1:e20. 10.1002/pcn5.20 PMC11114327 38868644

[8] Kanda Y . Investigation of the freely available easy‐to‐use software “EZR” for medical statistics. Bone Marrow Transplant. 2013;48:452–458. 23208313 10.1038/bmt.2012.244 PMC3590441

[9] Pedersen MT , Andersen TO , Clotworthy A , Jensen AK , Strandberg‐Larsen K , Rod NH , et al. Time trends in mental health indicators during the initial 16 months of the COVID‐19 pandemic in Denmark. BMC Psychiatry. 2022;22:25. 35012486 10.1186/s12888-021-03655-8 PMC8743441

[10] Varga TV , Bu F , Dissing AS , Elsenburg LK , Bustamante JJH , Matta J , et al. Loneliness, worries, anxiety, and precautionary behaviours in response to the COVID‐19 pandemic: a longitudinal analysis of 200,000 Western and Northern Europeans. Lancet Reg Health Eur. 2021;2:100020. 33870246 10.1016/j.lanepe.2020.100020 PMC8042675

