# Supplementary material for: Impact of the COVID‐19 pandemic on patients with bipolar disorder in Japan
Source: PCN Rep. 2023 Mar 2;2(1):e82. doi: 10.1002/pcn5.82 (PMC11114344; doi:10.1002/pcn5.82)
Supplement: Supplementary file 1 — Supporting information. [file PCN5-2-e82-s001.docx]

**Supporting Information**

1. The following three questions were used in this survey.

1）"To what extent has COVID-19 affected your medical condition?"

Options: very much worse, worse, a little worse, no impact, a little better, and better

2) "How did COVID-19 affect your symptoms? Please check the symptoms that got worse. (Multiple responses are allowed)" (To be answered by checking the options for specific symptoms.)

Options: □Depressive mood, □Feeling of exhilaration, □Low motivation, □Grandiosity, □Unmotivated to move, □Excessive spending, □Pressured speech, □Difficulty concentrating, □Low thinking ability, □Flight of ideas, □Suicidal ideation, □Irritability, □Self-injurious behavior, □Deviant behavior, □Sleeplessness, □Oversleeping, □Overeating, □Loss of appetite, □Other (Free answer)

3)"How has COVID-19 affected you? (Multiple responses are allowed)" (To be answered by checking the options for the specific life impact option.)

Options: □Unable to be hospitalized, □Had to be hospitalized, □Unable to be discharged, □Had to be discharged, □Unable to see visitors, □Extended hospital visits, □Increased waiting time at outpatient clinic, □Change of outpatient facility, □Loss of a family member or close people, □Infected with COVID-19 myself, □Family member or close people infected with COVID-19, □Increased stress due to not being able to go out, □Increased quarrels with family members, □Increased drinking or smoking, □Increased gaming time, □Increased time on social networking sites, □Other (Free answer)

2. The following table shows the number of respondents who reported that each symptom worsened. Depressive symptoms include "Depressive mood," "Low motivation," "Unmotivated to move," "Difficulty concentrating," "Low thinking ability," "Suicidal ideation," "Self -injurious behavior", "Sleeplessness", "Oversleeping", and "Loss of appetite". Manic symptoms included "Feeling of exhilaration", "Grandiosity", "Excessive spending", "Pressured speech", "Flight of ideas", "Irritability", and "Deviant behavior". For "neither" symptoms, "Overeating" and "Others (Free answer)" were included.

| Symptoms | Number of respondents who reported that each symptom worsened |
| --- | --- |
| Depressive mood | 64 |
| Feeling of exhilaration | 2 |
| Low motivation | 38 |
| Grandiosity | 2 |
| Unmotivated to move | 20 |
| Excessive spending | 17 |
| Pressured speech | 8 |
| Difficulty concentrating | 21 |
| Low thinking ability | 14 |
| Flight of ideas | 27 |
| Suicidal ideation | 19 |
| Irritability | 39 |
| Self-injurious behavior | 3 |
| Deviant behavior | 4 |
| Sleeplessness | 20 |
| Oversleeping | 18 |
| Overeating | 30 |
| Loss of appetite | 5 |
| Others (Free answer) | 17 |
| Depressive symptoms | 84 |
| Manic symptoms | 60 |
| Symptoms neither depressive nor manic | 46 |
| Any symptoms | 109 |
